# Comparison of combined intravenous and inhalation anesthesia and total intravenous anesthesia in laparoscopic surgery and the identification of predictive factors influencing the delayed recovery of neurocognitive function

**DOI:** 10.3389/fmed.2024.1353502

**Published:** 2024-03-25

**Authors:** Teng Song, Li-Jun Wu, Li Li

**Affiliations:** Department of Anesthesiology, Tongling Municipal Hospital, Tongling, China

**Keywords:** CIVIA, TIVA, laparoscopic surgery, delayed recovery of neurocognitive function, serum cytokines

## Abstract

**Background:**

Compare the anesthesia effects of combined intravenous and inhalation anesthesia (CIVIA) and total intravenous anesthesia (TIVA) in laparoscopic surgery. Furthermore, our objective is to examine the elements that contribute to the delay in postoperative recovery of neurocognitive function and anticipate the manifestation of delayed recovery by analyzing serum cytokines.

**Methods:**

The CIVIA group and the TIVA group both consisted of 130 patients who were scheduled to have elective major abdominal surgery through laparoscopy. The criteria taken into account by the observational and record-keeping study were the patients’ ages, sexes, body masses, heights, and the presence or absence of any preexisting problems. Both groups also had their anesthetic depth, duration, and per-unit-of-time muscle relaxant and analgesic dosages recorded. Finally, the length of each patient’s stay in the hospital as well as their overall length of stay were tracked. By using the Mini-Mental State Examination (MMSE) to measure cognitive function, we assessed the mental states of the subjects. Additionally, we wanted to identify any biomarkers that could be linked to postoperative cognitive decline or delays in neurocognitive recovery.

**Results:**

A total of 51 participants from the CIVIA group and 53 participants from the TIVA group satisfactorily completed the necessary neuropsychological exam for identifying delayed neurocognitive recovery at the study’s completion. In the initial data of the two groups, no significant discrepancies were found (*p* > 0.05). The CIVIA group exhibited noteworthy reductions in the quantity of administered analgesics and muscle relaxants per unit of time in comparison to the TIVA group (*p* < 0.05). In addition to this, the duration from the sevoflurane tank being closed to the extubation period demonstrated a significant reduction in the CIVIA group compared to the TIVA group (*p* < 0.05). Moreover, no statistically notable distinction was observed in terms of postoperative hospitalization duration and overall hospitalization duration among both groups (*p* > 0.05). According to the study, both the CIVIA group and the TIVA group had a total of 7 (13.72%) and 17 (32.07%) individuals, respectively, who met the criteria for neurocognitive delayed recovery (Odds Ratio: 0.336; 95% CI: 0.134–0.864; *p* = **0.026**). According to the research findings, it is indicated that there is a possibility for an increased presence of IL-6 in the bloodstream within 60 min following the incision made on the skin. This occurrence subsequently leads to the prolonged restoration of neurocognitive capabilities.

**Conclusion:**

The CIVIA technique outperforms the TIVA method in terms of overall assessment in the setting of laparoscopic surgery. It’s also important to remember that an increased blood IL-6 level during laparoscopy may operate as a separate risk factor for a delay in the restoration of neurocognitive function.

## 1 Introduction

Laparoscopic surgery, known for its advantages of minimal trauma, reduced bleeding, and faster postoperative recovery compared to open surgery (1–3). The concept of anesthesia and perioperative Enhanced Recovery After Surgery has brought forth new and significantly higher demands on anesthesia during laparoscopic surgery (4, 5). It not only requires ensuring safe anesthesia but also focuses on achieving faster recovery. Therefore, it is essential to carefully select appropriate anesthesia methods and drugs that can minimize the psychological trauma and physiological stress experienced by patients during anesthesia and the perioperative period (6). This approach can also help reduce the incidence of postoperative side effects, shorten hospital stays, lower hospitalization costs, facilitate the patient’s prompt recovery, and improve the hospital turnover rate (7–9).

Currently, there are three commonly used methods of general anesthesia for laparoscopic surgery: total intravenous anesthesia (TIVA), simple inhalation anesthesia, and combined intravenous and inhalation anesthesia (CIVIA) (10, 11). Among these methods, two general anesthetic drugs, sevoflurane, and propofol, are widely used in clinical practice (12). Sevoflurane is a new type of halogen inhalation anesthetic that offers advantages such as fast induction, rapid recovery, simple operation, and strong controllability (13). It does not irritate the respiratory tract and effectively prevents intraoperative awareness and intuitive reaction to the depth of anesthesia. It also has less impact on circulation and provides benefits such as analgesia, muscle relaxation, and organ protection (14). On the other hand, propofol is a short-acting intravenous general anesthetic known for its quick onset, strong efficacy, and minimal side effects. However, compared to sevoflurane, propofol has poorer analgesic and muscle relaxant effects, and it may cause injection pain. It also has a stronger impact on breathing and circulation and is relatively more expensive (15).

The establishment of pneumoperitoneum and the administration of anesthetic drugs during laparoscopic surgery can have an impact on the hemodynamic stability of patients and the smooth recovery of postoperative cognitive function (16). The use of anesthetic drugs can lead to a higher stimulation response, thereby significantly affecting cognitive function (17, 18). This trial tries to compare the doses of analgesics and muscle relaxants administered per unit of time under two different anesthesia methods, as well as evaluate the postoperative recovery quality system. Our experiment aimed to evaluate the effects of two anesthesia techniques, CIVIA (Sevoflurane-Remifentanil) and TIVA (Propofol-Remifentanil), on the delayed neurocognitive recovery of older individuals who are subjected to laparoscopic abdominal surgery. Additionally, we aimed to identify any blood biomarkers associated with delayed neurocognitive recovery.

## 2 Materials and methods

### 2.1 Clinical research grouping

This study, which took place from March 2019 to March 2023, followed a prospective, single-blind, randomized controlled trial design. The Tongling Municipal Hospital Institution Review Committee approved this research on January 10, 2019, under the approval number 2019-099-02. The study was registered on Clinicaltrials.gov.cn in November 2018 (trial registration number: NCT03856760). During the preoperative evaluation, informed permission was obtained from each person before participants were randomly assigned. The trial complied with the reporting standards set out by CONSORT, the Consolidated Standards of Reporting Trials. The principles set out in the Helsinki Declaration were followed throughout all processes. For this research, 130 patients who were scheduled to have major laparoscopic abdominal surgery were chosen. Using a computer-generated random number system to evenly split the patients into the CIVIA and TIVA groups. Our researcher gathered 42 control patients who were not having surgery to guarantee the validity and accuracy of the repeated neuropsychological tests.

In this study, the research statistician utilized a computer to generate random numbers without any constraints, following a simple randomization process. The randomization codes were sealed in sequentially numbered envelopes and delivered to the research coordinator by a research nurse a day before the surgery. The coordinator then informed the anesthesiologists of the group assignments for the patients, who were assigned to study groups based on these randomization codes. Patients were divided into the CIVIA group and the TIVA group in a 1: 1 ratio. Preoperative interviews, eligibility assessments, obtaining written informed consent, participant inclusion, and postoperative follow-up were all carried out by investigators who were not involved in perioperative patient care prior to the study and were trained in neuropsychological assessment. Both patients and investigators were blinded to the group assignments and were unable to conduct group studies.

### 2.2 Inclusion criteria and exclusion criteria

Inclusion criteria: (1) Individuals scheduled for elective laparoscopic abdominal surgery; (2) Participants without significant audiovisual disabilities and possessing reading capability.

Exclusion criteria: (1) patients have a life expectancy of fewer than 3 months or have serious underlying diseases; (2) patients with an MMSE score of fewer than 23 points; (3) patients with dementia; (4) patients currently taking drugs that affect the nervous system; (5) patients with alcohol addiction or long-term drug dependence; (6) patients who have previously been included; (7) patients with difficulty in follow-up; (8) patients with uncontrolled hypertension (over 180/100 mmHg).

### 2.3 Anesthesia and perioperative nursing

Following the patient’s entrance into the operating room, an intravenous channel was established to administer the necessary substances. Routine ECG monitoring was conducted, along with the monitoring of various factors such as the depth of anesthesia (BIS), end-tidal partial pressure of CO_2_ (PET CO_2_), the concentration of inhaled anesthetics, and muscle relaxation (TOF value). Before anesthesia induction, preoperative drugs including penehyclidine 0.5 mg and dezocine 0.1 mg/kg were administered intravenously.

TIVA group: first, administer oxygen and denitrification for 5 min. Then, turn on the muscle relaxation monitor and initiate 4 series of stimulation (TOF) modes while monitoring the contraction of the adductor hallucis muscle. A dosage of 0.05 mg/kg of midazolam, 0.2 μg/kg/min of remifentanil, 1–2 mg/kg of propofol, and 0.15 mg/kg of rocuronium injection were administered. Following endotracheal intubation, the patient was attached to the anesthesia machine to facilitate mechanical ventilation.

CIVIA group: The procedure begins by providing oxygen and denitrification for 5 min. The muscle relaxation monitor is then turned on for calibration. Once the calibration is completed, the doses of midazolam and remifentanil are the same as those used in the TIVA group. Initially, the patient inhales 6 L/min of pure oxygen and 8% concentration of sevoflurane. After the patient’s eyelash reflex disappears, the oxygen flow rate is reduced to 1 L/min and the concentration of sevoflurane is lowered to 4%. After tracheal intubation, a dosage of 0.15 mg/kg of cisatracurium is administered through injection.

Both groups maintained intraoperative anesthesia using different methods. The TIVA group utilized continuous pumping to administer propofol (2 ~ 5 mg·kg^−1^·h^−1^) and remifentanil (0.1 ~ 0.3 μg·kg^−1^·min-1). On the other hand, the CIVIA group employed inhalation of sevoflurane (1.0 MAC ~ 2.3 MAC) while administering the same dose of remifentanil as the TIVA group. To ensure the appropriate anesthesia level, the BIS value was maintained at 40–60 by adjusting the concentration of sevoflurane and the pumping speed of propofol. The pressure was maintained between 35 and 45 mmHg.

The administration of midazolam and cisatracurium ceased 30 min before the end of the operation. Intravenous prophylactic administration of azasetron (10 mg) was performed. The propofol infusion was stopped upon deflation of the abdomen or when sevoflurane inhalation was initiated. The remifentanil pump was discontinued after surgery. In the CIVIA group, anesthesia was discontinued and an artificial anesthesia gas adsorber was connected. The residual sevoflurane was eliminated using 6 L/min of fresh pure oxygen.

### 2.4 Observation index and data collection

The study recorded and observed the general conditions of two groups of patients, including age, gender, weight, height, and preoperative complications. The doses of analgesics (remifentanil) and muscle relaxants (cisatracurium) administered to each group of patients per unit of time were also observed and recorded. The following parameters were observed and recorded: the time from closing the sevoflurane tank to the extubation period, spontaneous breathing, spontaneous eye-opening, and removing the endotracheal tube. Additionally, the time when the two groups of patients entered the room (basic value Ta) and when the endotracheal tube was inserted (Tb), 5 min after endotracheal tube insertion (Tc), insufflation (Td), 5 min after insufflation (Te), end of skin suturing (Tf), endotracheal tube removal (Tg), and removal of BIS value 5 min after endotracheal tube (Th) were also observed and recorded. Furthermore, 20 min after extubation, the SAS and VAS were used to evaluate whether there was a difference in agitation and pain after extubation. Additionally, 5 mL of venous blood was drawn to detect biomarkers that could be associated with delayed recovery of neurocognitive function or postoperative cognitive dysfunction.

### 2.5 Enzyme-linked immunosorbent assay

Blood samples were taken from each participant, and left at room temperature for half an hour. Next, the samples were spun in a centrifuge to separate the liquid above, which was later kept for testing. The levels of IL-10, IL-1β, IL-6, TNF-a, VEGF, ICAM, TGF-β1, and APOE were determined using ELISA (19).

### 2.6 Neuropsychological assessment

Patients had cognitive testing the day before surgery and five to seven days thereafter. Similar to this, 6–9 days following the first exam, control individuals performed a second neuropsychological test. The delayed recovery of patients’ neurocognitive abilities was assessed using the following tests by the International Study of Postoperative Cognitive Dysfunction (ISPOCD): (1) Word Learning; (2) Word Recall; (3) Cognitive Flexibility; (4) Distraction; and (5) Work Memory, which was measured using an alphanumeric code. The neuropsychological assessment would be delayed for three days if the patient showed signs of disorientation during the exam. According to the criteria set forth by ISPOCD 1, the diagnosis of delayed neurocognitive recovery was made. In particular, patients were categorized as experiencing delayed neurocognitive recovery if their score on either of the two individual tests, represented by the Z-score or Z-combined value, was equal to or exceeded 1.96.

### 2.7 Statistical processing

The chi-square test or Fisher’s exact test were both used to analyze the categorical data, which were represented as frequencies (percentages). Depending on the distribution of the data, independent *t*-tests or Mann–Whitney U-tests were used to compare the continuous variables, which were provided as mean ± SD. To identify potential prognostic factors for delayed recovery of neurocognitive function, univariate logistic regression analysis was employed.

## 3 Results

### 3.1 Patient characteristics

**Figure 1:**
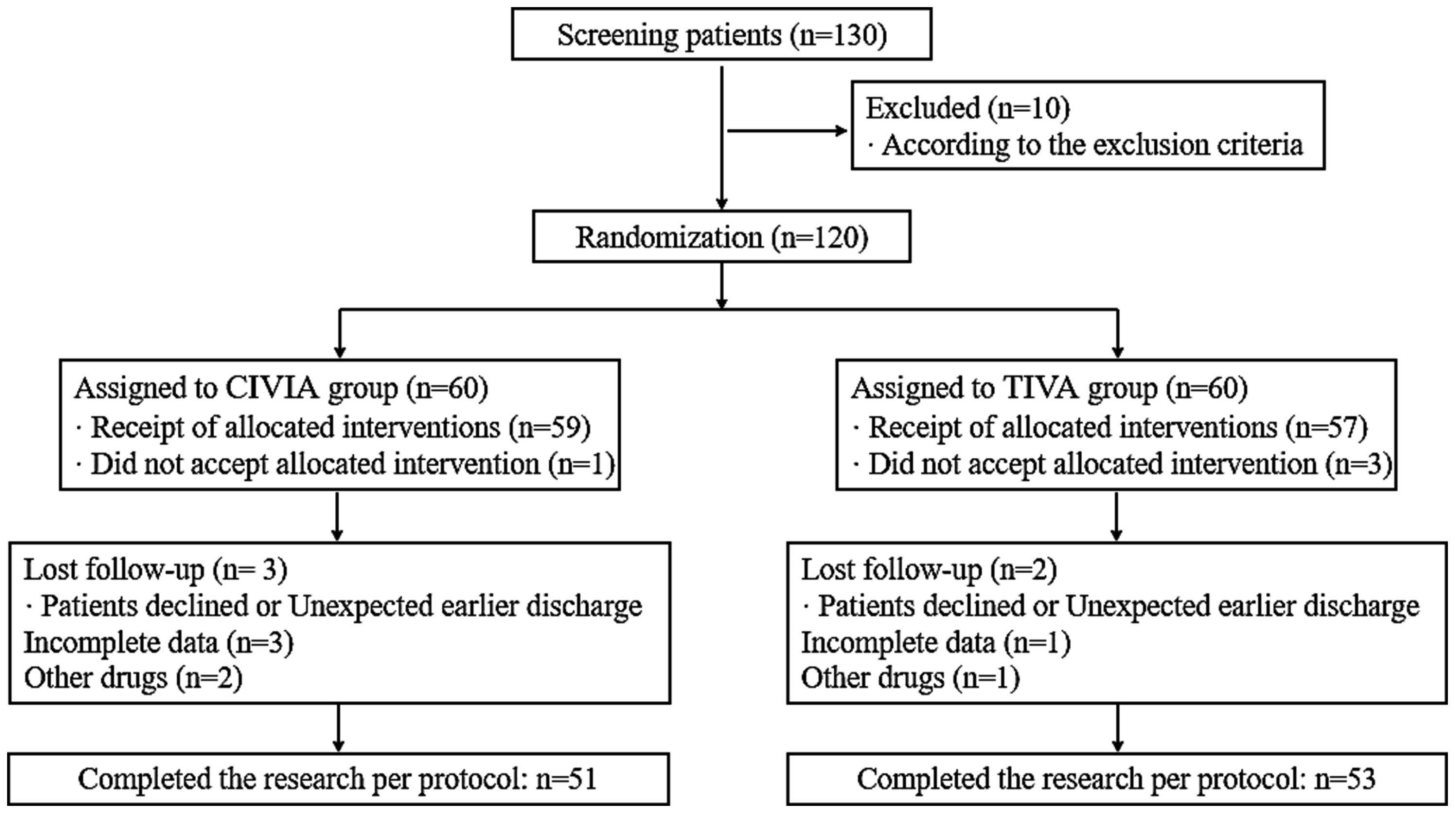
Flow chart of patient admission screening.

130 individuals in all were originally screened for the trial. The screening procedure was carried out in accordance with the established inclusion and exclusion criteria for this research as well as the statistical data collected from specific trials. A total of 51 patients in the CIVIA group and 53 patients in the TIVA group successfully completed the trial in line with the stated protocol (Figure 1).

No noteworthy disparities in the fundamental data were observed between the CIVIA and TIVA groups (*p* > 0.05). Moreover, there were no statistically significant variations in preoperative complications among the two groups (*p* > 0.05) (Table 1).

**Table 1:** Include the basic characteristics of the subjects ( $\bar{x}\pm s$ ).

	CIVIA group (*n*=51)	TIVA group (*n*=53)	*p*
Age, years	53.80±12.83	52.39±13.11	0.581
Sex			0.665
Female	31 (60.78)	30 (56.60)	
Male	20 (39.22)	23 (43.40)	
Body mass index, kg/m^2^	22.44±1.66	22.13±3.13	0.537
A mini-mental state examination score	29.02±0.91	29.0±0.89	0.912
Beck depression inventory score	2.57±1.30	2.56±1.25	0.991
State-anxiety inventory score	31.78±2.43	32.07±3.65	0.634
Trait-anxiety inventory score	31.0±2.37	31.30±3.64	0.618
Preoperative comorbidities			
Hypertension	15 (29.41)	16 (30.18)	0.931
Coronary artery disease	1 (1.96)	2 (3.77)	0.580
Diabetes mellitus	4 (7.8)	5 (9.4)	0.773
Arrhythmia	1 (1.96)	1 (1.89)	0.978
Chronic obstructive pulmonary disease	0 (0.0)	1 (1.88)	0.324

### 3.2 Comparative study on the dosage of anesthetics

**Figure 2:**
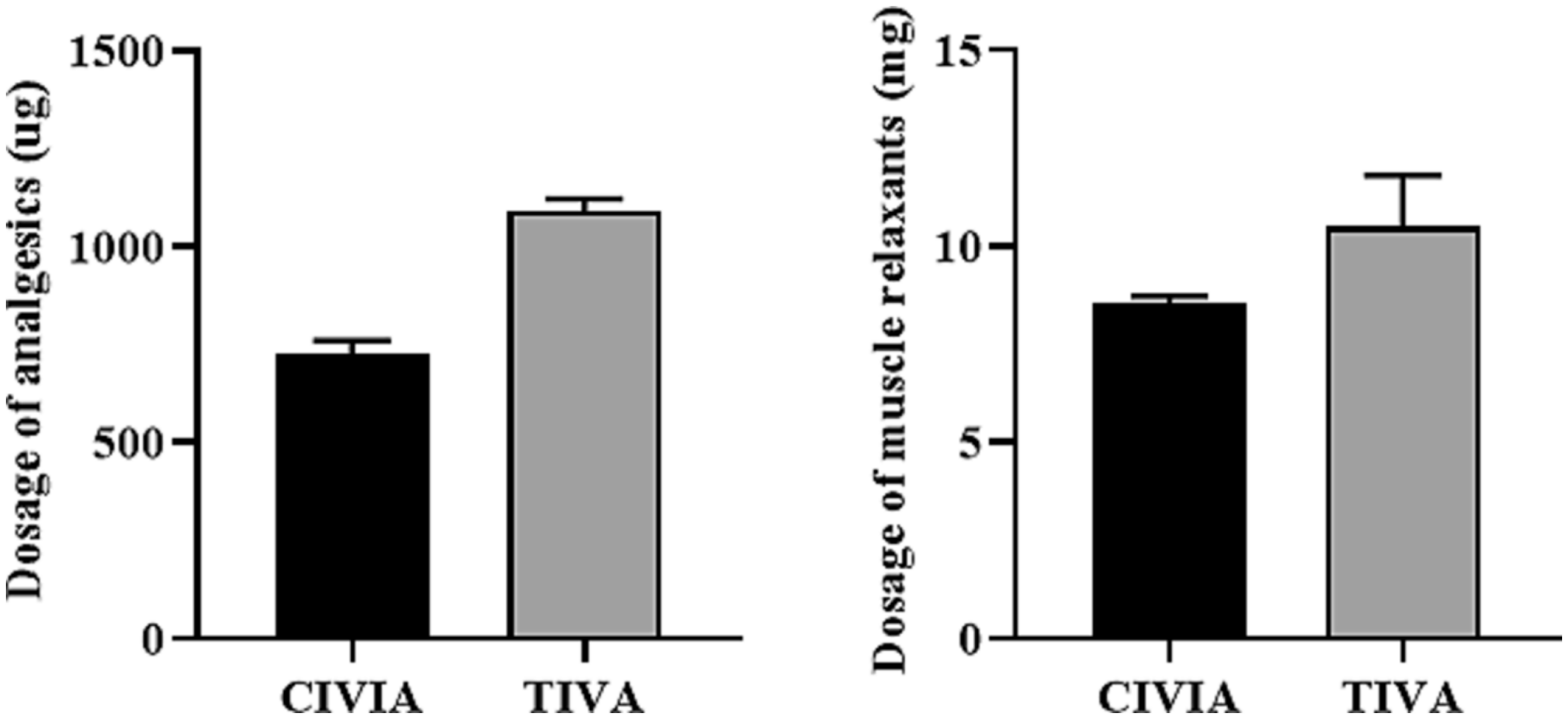
Comparison of the dosage of two anesthetic drugs per unit of time between the two groups.

According to the data presented in Table 2 and Figure 2, the CIVIA group required significantly lower doses of analgesics (remifentanil) and muscle relaxants (cisatracurium) per unit of time compared to the TIVA group (*p* < 0.001).

**Table 2:** Comparison of anesthetic dosage per unit time between the two groups ( $\bar{x}\pm s$ ).

	CIVIA group (*n*=51)	TIVA group (*n*=53)	*p*
The dose of painkillers (ug)	727.49±32.37	1090.86±30.65	**<0.001**
The dose of muscle relaxant (mg)	8.53±0.19	10.51±1.29	**<0.001**

The statistical significance was set at *p* < 0.05.

### 3.3 A comparative study on the awakening status of the two groups after anesthesia

**Figure 3:**
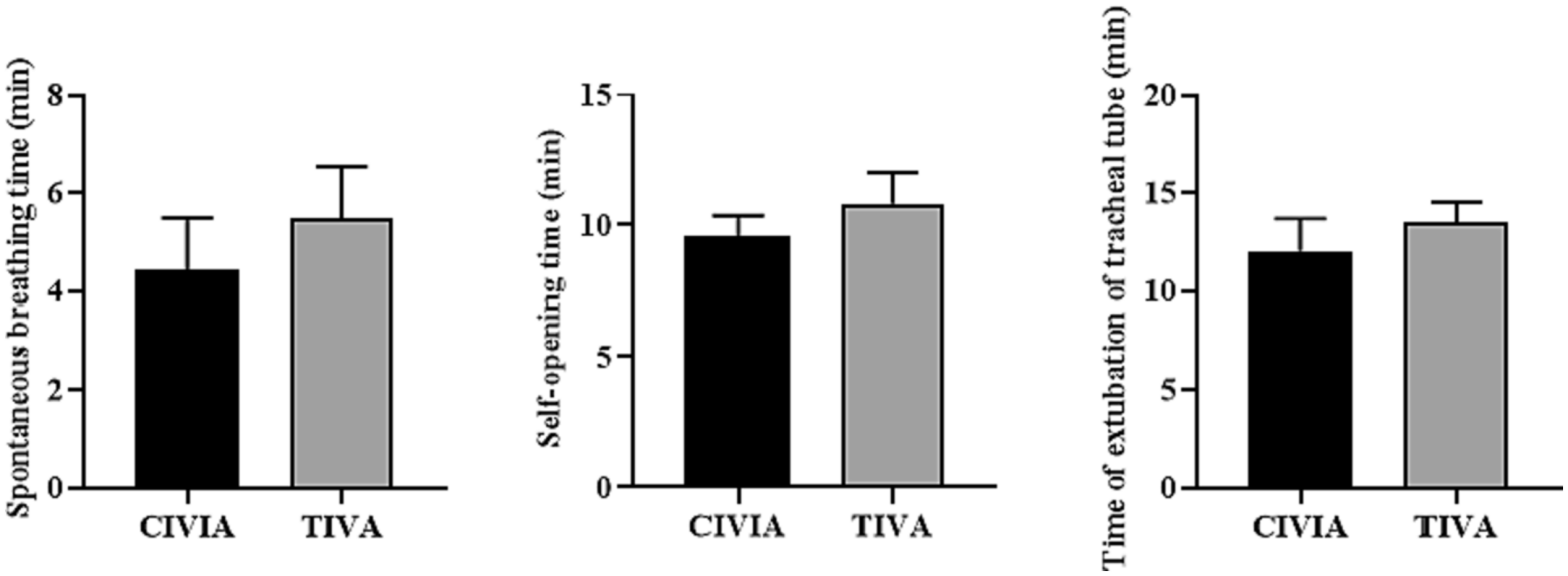
Comparison of recovery after operation between the two groups.

According to the results presented in Table 3 and Figure 3, it was observed that in both groups of patients, the time interval from closing the sevoflurane tank to the extubation period for spontaneous breathing, the time taken for spontaneous eye opening during the extubation period.

**Table 3:** Comparison of recovery after operation between the two groups ( $\bar{x}\pm s$ ).

	CIVIA group (*n*=51)	TIVA group (*n*=53)	*p*
Spontaneous breathing time	4.40±1.07	5.47±1.06	**<0.001**
Self-opening time	9.57±0.80	10.83±1.18	**<0.001**
Time of extubation of tracheal tube	12.02±1.64	13.49±1.01	**<0.001**

The statistical significance was set at *p* < 0.05.

### 3.4 Comparison of anesthetic depth at different time points between the two groups

**Figure 4:**
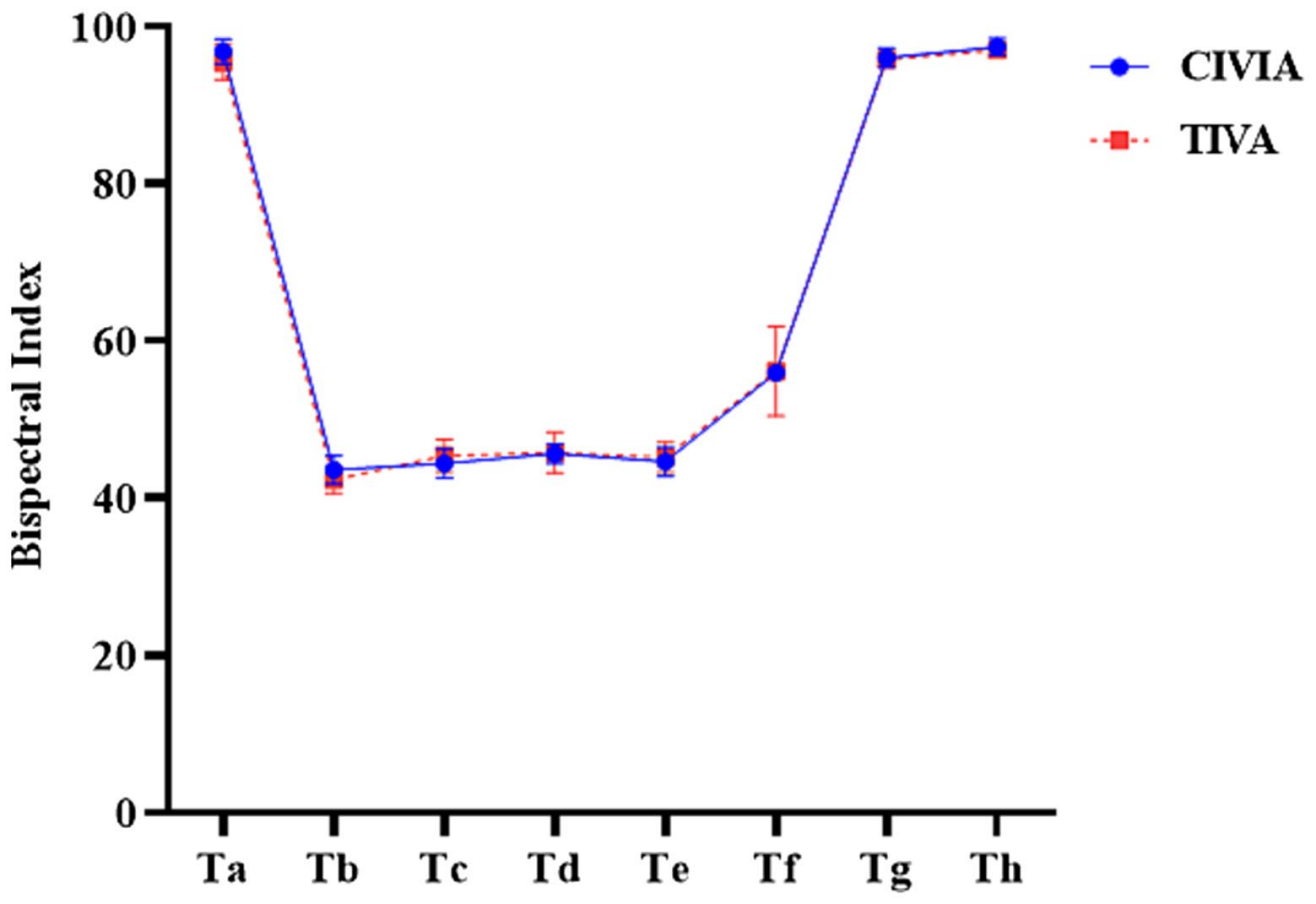
Comparison of anesthetic depth at different time points between the two groups.

The depth of anesthesia was compared between the CIVIA group and TIVA group at various time points (*p* > 0.05): when the two groups of patients entered the room (Ta) when the endotracheal tube was inserted (Tb), 5 min after the insertion of the endotracheal tube (Tc), when the pneumoperitoneum was inflated (Td), and 5 min after inflation of the pneumoperitoneum (Te). Additionally, the depth of anesthesia was compared at the end of skin suturing (Tf), at the time of removal of the endotracheal tube (Tg), and 5 min after removal of the endotracheal tube (Th) (Table 4; Figure 4).

**Table 4:** Comparison of anesthetic depth at different time points between the two groups ( $\bar{x}\pm s$ ).

BIS time	CIVIA group (*n*=51)	TIVA group (*n*=53)	*F* value	*p* value
Ta	96.80±1.58	95.45±2.28	0.20	0.56
Tb	43.60±1.81	42.30±1.75	0.49	0.60
Tc	44.40±1.87	45.40±2.07	1.12	0.28
Td	45.67±1.24	45.80±2.59	0.35	0.57
Te	44.60±1.87	45.20±1.92	0.40	0.55
Tf	55.64±1.48	56.10±5.71	0.67	0.23
Tg	96.00±1.19	95.80±0.99	1.06	0.28
Th	97.40±1.09	97.00±1.00	0.89	0.55

BIS: Bispectral Index. No statistical significance between the two groups.

### 3.5 A comparative study of pain and sedation and restlessness between the two groups after anesthesia

**Figure 5:**
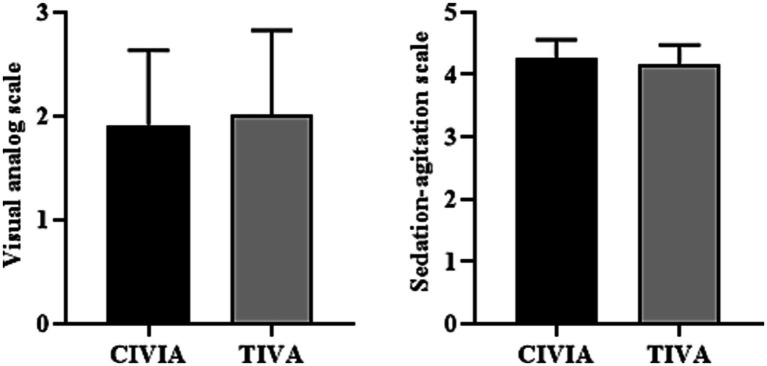
Comparison of pain score and sedation score between the two groups after extubation.

As shown in Table 5 and Figure 5, following extubation, the VAS scores between the two patient groups did not show a statistically significant difference (*p* > 0.05). Similarly, there was no statistically significant variance in the SAS scores between the two groups (*p* > 0.05).

**Table 5:** Comparison of pain score and sedation and restlessness score after extubation between the two groups ( $\bar{x}\pm s$ ).

Number	CIVIA group (*n*=51)	TIVA group (*n*=53)	*p*
VAS	1.91±0.72	2.01±0.81	0.520
SAS	4.26±0.29	4.16±0.31	0.098

### 3.6 Comparison of hospitalization days between two groups

**Figure 6:**
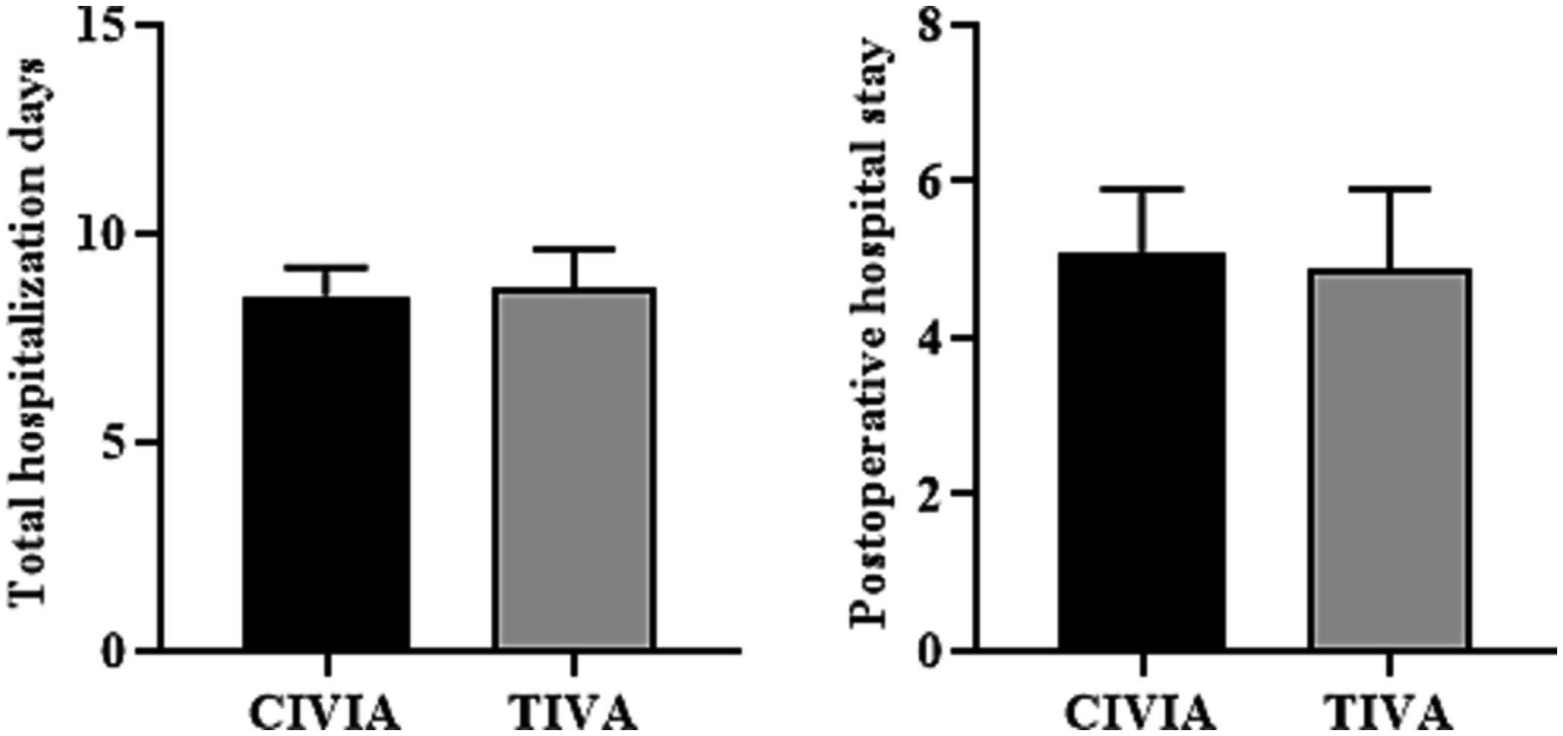
Comparison of hospitalization days between the two groups.

There was no difference in total hospitalization days and postoperative hospital days between the CIVIA and TIVA groups (*p* > 0.05) (Figure 6; Table 6).

**Table 6:** Comparison of hospitalization days between two groups ( $\bar{x}\pm s$ ).

Number	CIVIA group (*n*=51)	TIVA group (*n*=53)	*p*
Total hospitalization days	8.51±0.70	8.93±0.95	0.293
Postoperative hospital days	5.07±0.68	4.95±0.83	0.292

### 3.7 A comparative study of delayed neurocognitive recovery rate between the two groups

None of the patients experienced confusion during the predetermined test period, ensuring that the neuropsychological evaluation of each patient was not delayed. When analyzing the data according to the protocol, the control group exhibited a 4.8% delay rate in neurocognitive function recovery (2/42). In comparison, the CIVIA group and the TIVA group displayed significantly higher delay rates, with 13.72% (7/51) and 32.07% (17/53), respectively (*p* < 0.001). There is a variance in the rates of delayed neurocognitive recovery observed among the CIVIA and TIVA groups (*p* = 0.026) (Table 7).

**Table 7:** The occurrence of delayed recuperation of neurocognitive abilities among individuals administered various forms of general anesthetics.

	CIVIA group (*n*=51)	TIVA group (*n*=53)	Odds ratio (95% CI)	*p* value
Non-delayed neurocognitive recovery	44 (86.28)	36 (67.93)	0.336 (0.134-0.864)	**0.026**
Delayed neurocognitive recovery	7 (13.72)	17 (32.07)		

The number of patients (%) is represented as data. The chi-square test was used to calculate the *p* value.The statistical significance was set at *p* < 0.05.

### 3.8 A comparative study on the content of serum cytokines between two groups

**Figure 7:**
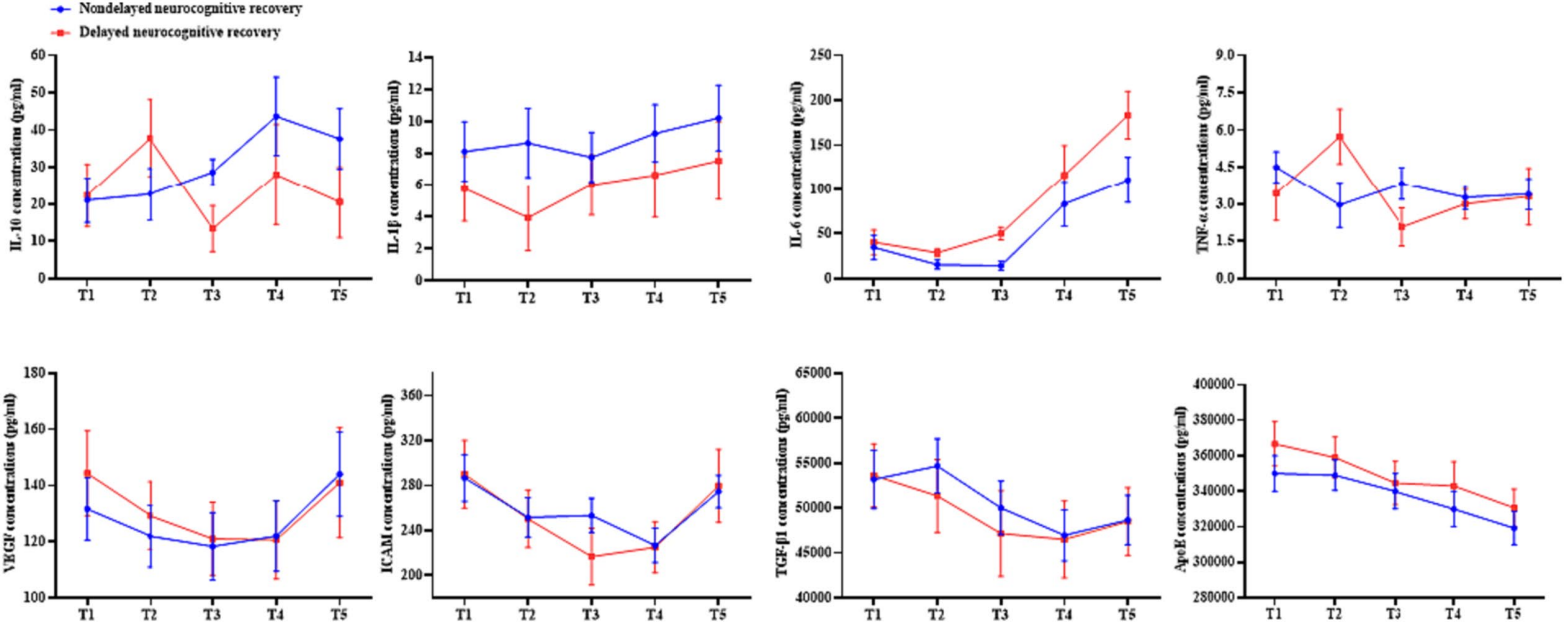
A comparison was conducted to assess the serum cytokine levels in patients with and without delayed neurocognitive recovery.

Table 8 and Figure 7 show that patients whose neurocognitive function recovery was slowed had higher levels of IL-6 than those whose recovery was not slowed (*p* = 0.018). It is also important to note that there was a significant interaction between time and group (*p* = 0.031), emphasizing the interaction between these two variables. Additional analysis revealed notable variations in IL-6 levels during T3 among individuals experiencing delayed neurocognitive recovery compared to those who did not encounter this issue (odds ratio, 1.06 [1.02 to 1.10]; *p* = 0.003). Although certain other blood factors demonstrated temporal alterations (VEGF, ICAM, TGF-β1, and ApoE), these factors did not differ at various time intervals between individuals with delayed neurocognitive recovery and those without such delay in cognitive recovery.

**Table 8:** Comparing serum cytokine levels in patients with or without delayed neurocognitive recovery between these two study cohorts.

Serum cytokines	Within-group		Between-group		Interaction	
	*F*-value	*p* value	*F*-value	*p* value	*F*-value	*p* value
IL-10	3.104	0.097	0.675	0.510	1.541	0.287
IL-1β	0.834	0.497	1.543	0.203	0.560	0.823
IL-6	21.129	**<0.001**	4.900	**0.018**	3.915	**0.031**
TNF-α	1.781	0.218	0.081	0.891	2.105	0.241
VEGF	5.117	**0.002**	0.062	0.829	0.409	0.787
ICAM	8.534	**<0.001**	0.084	0.810	1.615	0.204
TGF-β1	3.760	**0.006**	0.151	0.871	0.432	0.998
ApoE	5.230	**0.001**	0.645	0.484	0.493	0.802

The statistical significance was set at *p* < 0.05.

### 3.9 Prognostic factors of delayed recovery of neurocognitive function

Through the examination of univariate logistic regression, various risk factors that exerted an impact on the restoration of neurocognitive abilities were identified. These factors included age (*p* = 0.040), Mini-Mental State Examination score (*p* < 0.001), State-anxiety inventory score (*p* = 0.014), Trait-anxiety inventory score (*p* = 0.003), anesthesia time (*p* = 0.006), hospitalization time (*p* < 0.001), and IL-6 concentration at T3 (*p* = 0.003). The results demonstrate that increased age, longer duration of anesthesia, prolonged hospitalization, and elevated levels of IL-6 at T3 are correlated with a heightened occurrence of delayed neurocognitive restoration (Table 9).

**Table 9:** A potential predictor analysis of neurocognitive recovery delay using univariate logistic regression.

	Delayed neurocognitive recovery (*n* = 24)	Nondelayed neurocognitive recovery (*n* = 80)	Univariate Odds Ratio (95% CI)	*p* value
Age, years	66.6±5.34	63.65±8.67	1.07 (1.03–1.12)	**0.040**
Sex				0.635
Female	6 (25.0)	24 (30.0)	Reference	
Male	18 (75.0)	56 (70.0)	1.28 (0.47-3.67)	
Body mass index, kg/m^2^	22.38±1.62	22.24±2.73	1.00 (0.97–1.03)	0.811
A mini-mental state examination score	26.5±1.64	27.78±1.42	0.82 (0.72–0.93)	**<0.001**
Trait-anxiety inventory score	30.45±2.41	32.68±3.33	0.99 (0.97–1.02)	**0.003**
Beck depression inventory score	2.5±1.31	2.58±1.25	1.04 (0.98–1.10)	0.768
State-anxiety inventory score	30.58±2.60	32.33±3.14	0.98 (0.96–1.01)	**0.014**
American Society of Anesthesiologist’s physical status				
I	1 (4.2)	8 (10.0)	Reference	0.372
II	17 (70.8)	64 (80.0)	1.35 (0.50–3.61)	0.342
III	6 (25.0)	8 (10.0)	3.65 (1.24–10.7)	0.059
Interleukin-6 concentration at T3, pg/ml	52.12±5.41	12.51±3.41	1.05 (1.02–1.09)	**0.003**
Duration of hospitalization, day	10±2.29	8±0.88	1.06 (1.02–1.09)	**<0.001**
Anesthesia time, h	4.5±0.67	4.13±0.52	1.08 (0.94–1.24)	**0.006**

The statistical significance was set at *p* < 0.05.

## 4 Discussion

Laparoscopic surgery necessitates swift anesthesia induction, precise anesthesia depth control, and steady intraoperative hemodynamics. Additionally, it demands prompt postoperative recovery devoid of complications like respiratory depression, nausea, and vomiting (20, 21). This study explores the use of dual short-acting anesthetics in TIVA and CIVIA protocols to assess if the selection of anesthetic agents and methods influences the risk of delayed neurocognitive recovery or postoperative cognitive dysfunction.

After inhalation for anesthesia, sevoflurane is rapidly metabolized, facilitating the quick recovery of patients. Additionally, it offers convenient operation, ease of use, and control. Research has demonstrated that sevoflurane has several advantages over propofol, including minimal respiratory depression, simple anesthesia depth control, and safer postoperative recovery (22–24). This study found that the CIVIA group, which used sevoflurane for anesthesia induction and maintenance, had significantly shorter times for closing the sevoflurane tank to extubation, spontaneous eye-opening, and extubation of the endotracheal tube compared to the TIVA group. These findings indicate that patients who receive sevoflurane anesthesia can quickly regain consciousness after surgery, aligning with the results of previous analyses on the effects and economic benefits of different sevoflurane and propofol anesthesia schemes in laparoscopic surgery (25). Furthermore, it was observed in clinical application research that sevoflurane, when reaching a certain intensity, can induce muscle relaxation, with the degree of muscle relaxation increasing over time (26). The study also revealed that the CIVIA group required significantly lower doses of muscle relaxants per unit of time compared to the TIVA group. Both anesthesia methods demonstrated satisfactory analgesic effects, with sevoflurane potentially exhibiting a stronger analgesic effect than propofol. Notably, the CIVIA group had a significantly lower dosage of analgesics per unit of time than the TIVA group, suggesting that sevoflurane possesses analgesic properties that may surpass those of propofol.

Numerous clinical studies have investigated the impact of anesthetic agent choice and mode of anesthesia on the delay of neurocognitive recovery. Research findings indicate that the utilization of propofol as an anesthetic agent may result in a decreased occurrence of delayed neurocognitive recuperation in comparison to the use of volatile anesthetics (27). However, most previous studies have only utilized declines in Mini-Mental State Examination scores as a diagnostic measure for delayed neurocognitive recovery (28). The main objective of our investigation was to evaluate whether variations existed in the occurrence of postponed neurocognitive recuperation among individuals who received general anesthesia predominantly sustained with CIVIA and TIVA, particularly for laparoscopic procedures. Future studies should include multi-center, multi-sample studies to investigate potential differences in the incidence of delayed neurocognitive recovery between patients maintained under general anesthesia with CIVIA and TIVA.

As seen by their lower MMSE scores compared to individuals who did not have delayed neurocognitive recovery, it is interesting to note that these patients had worse cognitive performance (29). Additionally, these individuals spent longer in the hospital, which is consistent with other findings (30, 31). Age, Mini-mental State Examination score, State-anxiety inventory score, Trait-anxiety inventory score, duration of anesthesia, and length of hospital stay were found to be significant predictors of delayed neurocognitive recovery in the analysis of Univariate Logistic regression. One hour after the surgical incision, elevated blood levels of interleukin-6 have been shown to be a risk factor for a delayed neuropsychological recovery. Interleukin-6 levels rise after surgery, according to a number of studies (32, 33). According to these results, postoperatively elevated blood IL-6 levels were seen in both patients with and without delayed neurocognitive recovery. Patients who had a delayed neurocognitive recovery, however, had greater blood IL-6 levels than those who did not. This provides preliminary support for the idea that exacerbated inflammation may be a pathogenic factor in human neurocognitive recovery that is delayed (34). Within 24 h of surgery, no significant changes in blood levels of IL-1β, IL-10, or tumor necrosis factor were seen. These findings suggest that among surgical patients, some cytokines, such as interleukin-6, are induced by surgery.

## 5 Conclusion

Our study demonstrates that patients undergoing laparoscopic abdominal surgery under TIVA exhibit a greater likelihood of experiencing delayed neurocognitive recovery compared to those under CIVIA. Additionally, we have established that elevated serum levels of interleukin-6 serve as an independent risk factor for delayed neurocognitive recovery. These results offer valuable clinical insights into the connection between inflammation and delayed neurocognitive recovery.

## Data availability statement

The original contributions presented in the study are included in the article/supplementary material, further inquiries can be directed to the corresponding author.

## Ethics statement

The studies involving humans were approved by the Institutional Review Board of Tongling Municipal Hospital on 10/1/2019 (approval number: 2019-099-02). All the experiments of this study were conducted in accordance to the relevant guidelines and regulations or in accordance to the Declaration of Helsinki. Informed consent was obtained from all subjects involved in the study. Written informed consent was obtained from all participants. The studies were conducted in accordance with the local legislation and institutional requirements. The participants provided their written informed consent to participate in this study. Written informed consent was obtained from the individual(s) for the publication of any potentially identifiable images or data included in this article.

## Author contributions

TS: Conceptualization, Software, Writing – review & editing. L-JW: Investigation, Supervision, Writing – review & editing. LL: Writing – original draft, Writing – review & editing.
